# Complicated spontaneous pneumoparotid mimicking a neck mass in a child with Down’s syndrome

**DOI:** 10.4102/sajr.v24i1.1883

**Published:** 2020-07-13

**Authors:** Sheree C. Gray, Jacobus A. Pienaar, Zelia Sofianos, Jacob Varghese

**Affiliations:** 1 Department of Radiology, Faculty of Radiology, Klerksdorp/Tshepong Hospital Complex, Klerksdorp, South Africa; 2 Department of Radiology, Faculty of Radiology, University of the Witwatersrand, Johannesburg, South Africa

**Keywords:** Pneumoparotid, Spontaneous, Self-induced pneumoparotid, Stensen’s duct, Paediatric population

## Abstract

Spontaneous or self-induced pneumoparotid, which usually manifests as acute unilateral gland enlargement, is caused by insufflation of air from the oral cavity via Stensen’s duct. A 9-year-old patient, with known Down’s syndrome, presented with a progressively enlarging, painless, spontaneous, left neck mass. Computed tomography showed features consistent with pneumoparotid, without underlying associated pathology. Identification of true cases of spontaneous pneumoparotid is crucial, as these require a holistic management approach to prevent recurrence and complications.

## Introduction

Self-induced or spontaneous pneumoparotid, usually manifesting as acute unilateral enlargement of the parotid gland, is caused by the insufflation of air from the oral cavity via Stensen’s (parotid) duct. It is a rare but well-described condition and should be considered in cases of unexplained parotid swelling. Occasionally, this phenomenon may only be detected on investigation of associated complications, making clinical diagnosis challenging. Often, the condition will only be diagnosed after radiological imaging. Given that conservative management and behavioural modification is the mainstay of care in uncomplicated cases, accurate and timely radiological diagnosis is essential to prevent unnecessary surgical intervention and guide appropriate management of subsequent complications.

## Case presentation

A 9-year-old female patient, known with Trisomy 21, presented to the Ear, Nose and Throat (ENT) clinic with a large left-sided neck mass; this mass was progressively enlarging over a period of 1 month and began as a painless and spontaneous swelling. Other than the pre-existing genetic anomaly, the child was healthy and thriving and had no documented medical co-morbidities, nor any history of recent dental procedures or hospitalisations. Clinical examination revealed a firm, non-tender mass lesion on the left side of the neck, extending from below the pinna of the left ear to the submandibular region. Systemic examination was otherwise unremarkable, with no fever or other features of septicaemia. The overlying skin was intact, and no lymphadenopathy was palpable. Perfunctory ENT and oral examination were normal. Fine needle aspiration of the mass was attempted at the ENT clinic whilst awaiting laboratory results and radiological investigations, in an attempt to expedite the diagnostic process and to exclude sepsis. Only air was obtained. There was temporary subsidence of the mass post-aspiration, but it reformed within 24 h. Clinical assessment clearly suggested local surgical emphysema. Expeditious delineation of the cause and extent of the process was crucial, prompting the referring clinician to request a computed tomography (CT) scan of the head and neck to further delineate the lesion. Whilst awaiting imaging in the Radiology Department, the patient was observed to be voluntarily blowing up her cheeks and making other uncharacteristic buccal and facial movements.

**FIGURE 1:**
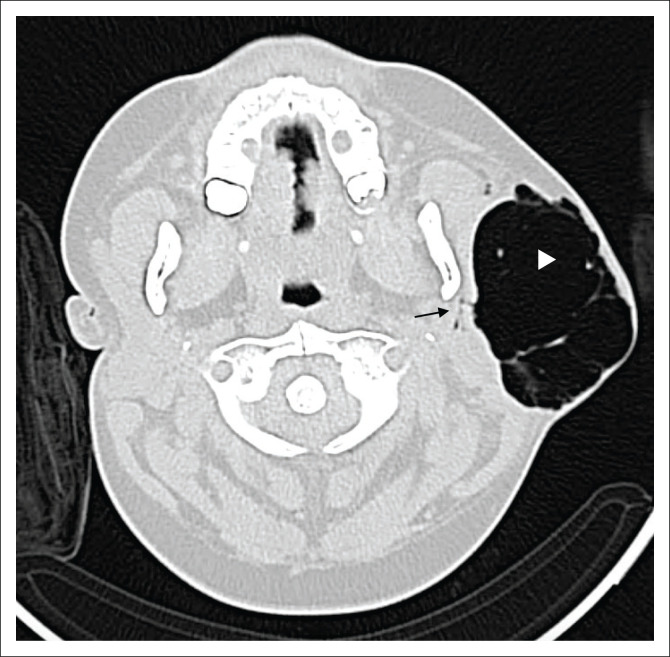
Axial non-contrasted computed tomography demonstrates a large, multiseptated focal collection of gas in the subcutaneous tissue overlying the left parotid gland (white arrowhead). Underlying communication with branching gas pattern in the ductal system of the left parotid gland is shown (black arrow). No gas is present in the parapharyngeal fat space or deep neck spaces.

**FIGURE 2:**
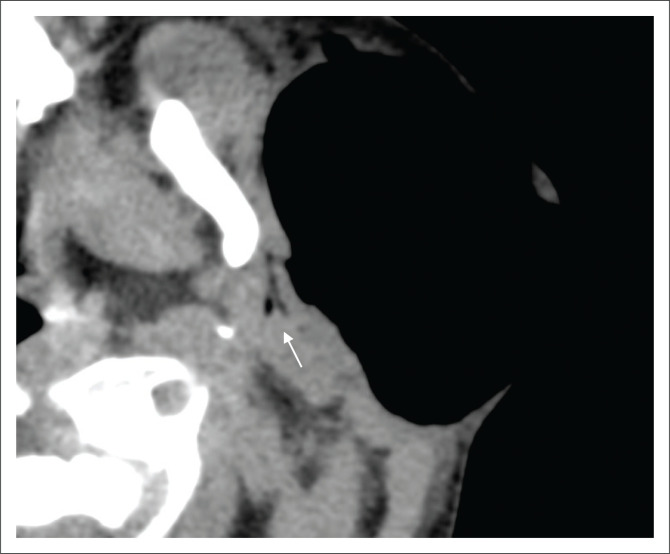
Magnified axial non-contrasted computed tomography with soft tissue window again demonstrates the gas-filled branching parotid ductal pattern (white arrow). The left parotid gland appears otherwise morphologically normal, with no features of inflammation.

**FIGURE 3:**
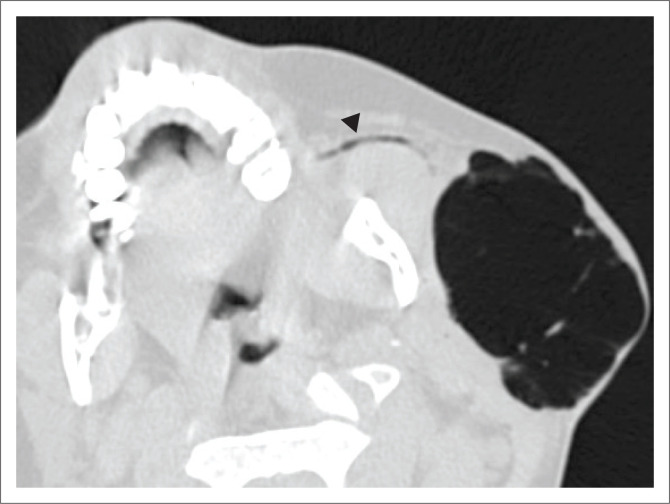
Magnified axial non-contrasted computed tomography demonstrating gas in the mildly distended left Stenson’s (parotid) duct (black arrowhead).

Non-contrast CT of the head and neck (performed with the patient under conscious sedation) demonstrated a circumscribed focus of subcutaneous surgical emphysema overlying the left parotid gland (Figure 1). No complex fluid content or surrounding inflammatory changes were present to suggest abscess formation. Gas was noted within the ductal system of the parotid gland (Figure 2) as well as intraductal gas following the trajectory of the left Stensen’s duct (Figure 3). The right parotid gland and associated ductal system were normal.

No fluid collections or masses of the head or neck were observed. Furthermore, there was no gas identified in the floor of the mouth, parapharyngeal spaces or deep soft tissue spaces of the neck. No foreign bodies or sialolithiasis was evident. The oral cavity, pharynx, larynx and trachea were unremarkable. The visualised lung apices and upper mediastinum showed no pneumothorax or pneumomediastinum.

These imaging features are consistent with pneumoparotid, complicated by capsular perforation and overlying localised subcutaneous surgical emphysema, in the absence of underlying associated pathology.

### Ethical considerations

This article followed all ethical standards for a research without direct contact with human or animal subjects.

## Discussion

Pneumoparotid refers to the abnormal presence of air within the ducts and acini of the parotid gland and occurs when intra-oral pressure overwhelms the usual protective mechanism of the meatus of Stensen’s duct.^1^

Since the initial report of air in the parotid gland by Hyrtl in 1865, a spectrum of different terminology has been used to denote the condition, including ‘pneumoparotitis’, ‘pneumoparotiditis’, ‘pneumosialoadenitis’, ‘wind parotiditis’, ‘anesthetic or surgical mumps’ and ‘pneumatocele glandulae parotis’. Current consensus is that the term ‘pneumoparotid’ should be used in the absence of inflammatory changes of the gland and ‘pneumoparotitis’ should be used in the presence thereof.^2^

The pathogenesis of pneumoparotid starts with an abnormal increase in pressure in the oral cavity, which is normally as low as 2 mmHg – 3 mmHg. This rise in oral pressure leads to the reflux of air into Stensen’s duct and subsequently into the ductal system of the parotid gland. If the intraductal pressures become sufficiently high, breakdown of glandular tissue can occur, leading to leakage of air into the surrounding soft tissue spaces and surgical emphysema if perforation of the parotid fascia occurs. Infrequent complications that can develop in the setting of large volume or continuous leakage of air from the gland are pneumomediastinum, pneumothorax and airway obstruction because of focal collections of air. In addition, oral bacteria may also reflux, predisposing to recurrent episodes of parotitis, termed ‘pneumoparotitis’, which can lead to ductal ectasia if chronic, or associated abscess formation.^2,3,4^

The aetiology of pneumoparotid is commonly divided into occupational and non-occupational categories. Occupational causes are most commonly glassblowing or the playing of wind instruments (which can increase the intra-oral pressure to more than 150 mmHg), as well as during rapid compression manoeuvres in scuba divers. Non-occupational causes are commonly iatrogenic, associated with dental procedures (in which devices powered by compressed air are used), and following anaesthesia, if the patient coughs during extubation while simultaneously receiving positive pressure ventilation. Cases of pneumoparotid post-pulmonary function testing have also been described. Pneumoparotid as a consequence of facial trauma is commonly encountered.^3,4^

A phenomenon which is much less commonly documented is spontaneous pneumoparotid or self-induced pneumoparotid. In rare instances, this may be identified as the underlying primary cause and as a result of auto-insufflation of the gland. In the paediatric population, auto-insufflation is the most common cause of pneumoparotid.^4^ Spontaneous pneumoparotid has been described after innocuous and common activities such as blowing up balloons, chewing gum, nose blowing or coughing, which are seen more frequently in individuals with underlying pathology such as cystic fibrosis. Self-induced pneumoparotid has also been reported in conjunction with psychiatric and psychosomatic disorders or because of an unintentional adjustment reaction to achieve secondary gains (such as avoiding school), especially in adolescents.^2,3,4^

Usually, inherent protective anatomical mechanisms are in place to prevent reflux from the oral cavity into the parotid glands, including mucosal folds that seal off the ductal orifice, the small diameter of the ductal orifice itself and contraction of the buccinator muscle to close the duct. It has been proposed that in children Stensen’s duct has a more perpendicular insertion into the buccal mucosa than in adults, in whom the angle of insertion is more acute posteriorly. This allows a buccal mucosal fold to provide improved protection in adults against the retrograde air travelling in Stensen’s duct when intra-oral pressure is increased.^5^ Underlying anatomical abnormalities such as ectasia of the parotid duct, buccinator muscle weakness or masseter hypertrophy may predispose to the development of pneumoparotid.^3^

Mild cases of spontaneous pneumoparotid may go completely unnoticed unless complications occur. The condition may have a chronic course with intermittent, short-lived episodes of painless or painful parotid swelling, which may become evident on clinical history. Visualisation of an oedematous Stensen’s papilla, air bubbles coming from Stensen’s duct or foamy saliva after parotid gland massage and crepitus upon cheek or gland palpation may be identified with careful and thorough clinical examination.^3,4^

Imaging can establish the diagnosis and delineate associated complications. Plain film and fluoroscopic studies will likely be normal or demonstrate only complications such as surgical emphysema or pneumothorax and may not be sufficient to delineate the underlying cause.^2^ Ultrasound, as a first line of radiological investigation in the paediatric population, is a valid consideration because it will demonstrate air bubbles as intraductal, mobile, comet-tail artefacts. High-frequency ultrasound may be useful in delineating potential associated ductal alterations and calculi. Colour Doppler may demonstrate hypervascularity with parotitis.^6^ However, the extent, distribution and origin of the gas, as well as concurrent deep compartment complications, may not be readily apparent, and interpretation of the study may somewhat depend on the operator. Non-contrast CT is especially useful in this setting to demonstrate both air in the parotid gland and associated complications, whilst also ruling out underlying sialolithiasis. Magnetic resonance imagining (MRI) may show associated signal changes in the parotid gland related to complications, but air may not be easily identified.^6^ Chronic pneumoparotitis is usually associated with ductal dilatation and stricturing because of associated episodes of bacterial superinfection. In this instance, sialography typically shows a markedly dilated Stensen’s duct, but the use of invasive tests is discouraged because of potential complications.^3^

In most cases of spontaneous uncomplicated acute pneumoparotid, salivary gland enlargement resolves spontaneously within 1–3 days, with conservative management yielding good results. Yet, in paediatric cases – as described above – treatment of the underlying cause for the abnormal buccal manoeuvres which result in the auto-insufflation may be very challenging as the patient’s understanding may somewhat be limited.^3,4^

Alternate treatment options such as antibiotics, anti-inflammatory drugs, glandular massage or sialogogues are usually reserved for cases with confirmed complications, such as sialadenitis. In the event that conservative measures fail, surgical intervention should be considered. Several surgical treatment options have been suggested for chronic and recurrent pneumoparotid, such as transposition of Stensen’s duct to the tonsillar fossa, ligation of the duct or superficial parotidectomy if chronic or recurrent infection occurs.^2,3^

## Conclusions

Pneumoparotid and its associated complications can be accurately diagnosed radiologically, and CT should be considered as the initial investigation of choice. However, especially in the paediatric population, detailed history-taking and clinical assessment remain imperative to rule out any underlying causes. This allows for the identification of true cases of spontaneous pneumoparotid, which require a more holistic management approach based on behavioural modification and psychotherapy.
